# Reliability, acceptability, validity and responsiveness of the CHU9D and PedsQL in the measurement of quality of life in children and adolescents with overweight and obesity

**DOI:** 10.1038/s41366-023-01305-5

**Published:** 2023-04-18

**Authors:** Alison Hayes, Rakhee Raghunandan, Anagha Killedar, Sarah Smith, Erin Cvejic, Martin Howell, Stavros Petrou, Emily Lancsar, Germaine Wong, Jonathan Craig, Kirsten Howard

**Affiliations:** 1 University of Sydney School of Public Health, Sydney, NSW Australia; 2 Menzies Centre for Health Policy and Economics, University of Sydney, Sydney, NSW Australia; 3 Department of Health Services Research and Policy, London School of Hygiene & Tropical Medicine, London, UK; 4 Nuffield Department of Primary Care Health Sciences, University of Oxford, Oxford, UK; 5 Department of Health Services Research and Policy, Australia National University, Canberra, ACT Australia; 6 College of Medicine and Public Health, Flinders University, Adelaide, SA Australia

**Keywords:** Paediatrics, Signs and symptoms

## Abstract

**Background:**

The Paediatric Quality of life Inventory (PedsQL^TM^) Generic Core Scales and the Child Health Utilities 9 Dimensions (CHU9D) are two paediatric health-related quality of life (HRQoL) measures commonly used in overweight and obesity research. However, no studies have comprehensively established the psychometric properties of these instruments in the context of paediatric overweight and obesity. The aim of this study was to assess the reliability, acceptability, validity and responsiveness of the PedsQL and the CHU9D in the measurement of HRQoL among children and adolescents living with overweight and obesity.

**Subjects/Methods:**

Subjects were 6544 child participants of the Longitudinal Study of Australian Children, with up to 3 repeated measures of PedsQL and CHU9D and aged between 10 and 17 years. Weight and height were measured objectively by trained operators, and weight status determined using World Health Organisation growth standards. We examined reliability, acceptability, known group and convergent validity and responsiveness, using recognised methods.

**Results:**

Both PedsQL and CHU9D demonstrated good internal consistency reliability, and high acceptability. Neither instrument showed strong convergent validity, but PedsQL appears to be superior to the CHU9D in known groups validity and responsiveness. Compared with healthy weight, mean (95%CI) differences in PedsQL scores for children with obesity were: boys −5.6 (−6.2, −4.4); girls −6.7 (−8.1, −5.4) and differences in CHU9D utility were: boys −0.02 (−0.034, −0.006); girls −0.035 (−0.054, −0.015). Differences in scores for overweight compared with healthy weight were: PedsQL boys −2.2 (−3.0, −1.4) and girls −1.3 (−2.0, −0.6) and CHU9D boys: no significant difference; girls −0.014 (−0.026, −0.003).

**Conclusion:**

PedsQL and CHU9D overall demonstrated good psychometric properties, supporting their use in measuring HRQoL in paediatric overweight and obesity. CHU9D had poorer responsiveness and did not discriminate between overweight and healthy weight in boys, which may limit its use in economic evaluation.

## Introduction

Overweight and obesity are pervasive conditions in childhood, with prevalence in developed countries of 24% in boys and 23% in girls [1]. In Australia, in 2018, 25% of children and adolescents aged 2–17 years were in an overweight or obese weight range. This is concerning due to the associations during childhood with elevated cardiometabolic risk factors, [2] type 2 diabetes [3] asthma [4], musculoskeletal pain [5] and depression [6]. Beyond clinical outcomes, there is evidence that patient reported outcome measures (PROMs) such as functional health status or health-related quality of life (HRQoL) are also impacted by weight status in childhood [7–9]. HRQoL measures are important aspects of patient-centred care and can inform economic evaluations and funding decisions regarding prevention and treatment [10]. HRQoL may be condition specific or generic, with the advantage that generic measures can be used across a range of childhood diseases or conditions. When used in economic evaluations HRQoL measures also require a preference-based value set (or utilities) to calculate quality-adjusted life-years (QALYs).

Two paediatric HRQoL measures commonly used in overweight and obesity research are the Paediatric Quality of life Inventory (PedsQL^TM^), the most widely used generic paediatric HRQoL measure [11], and the Child Health Utilities 9 Dimensions (CHU9D)[12], the first ‘preference-based’ measure designed specifically for children and with their involvement. PedsQL and CHU9D are different instruments with different purposes: the former measuring general quality of life and the latter measuring utilities for economic evaluation. There is consistent evidence that childhood overweight and obesity are associated with impaired HRQoL when measured with the PedsQL [9] which is the most frequently used HRQoL measure in the context of obesity [13]. Similarly, the CHU9D is widely used in cost-utility analyses of obesity prevention, yet the evidence for reduced HRQoL associated with overweight and obesity, using this measure, has been mixed and may depend on age and context [14–16]. The underlying pathways between weight and child HRQoL have not been established, but may be due to obesity-related co-morbidities [7], and reduced psychosocial and physical health [7, 8, 17].

Psychometric evaluation is important for establishing that an instrument is ‘fit for purpose’ in the measurement of HRQoL. The psychometric properties of the PedsQL have been established in general child and adolescent populations in the USA, [18] Netherlands [19] and Greece, [20] thus validating its measurement properties in population health. Similarly, the feasibility, reliability and validity of the CHU9D has been established in general child populations in Denmark [21], Sweden [22], Australia [23] and China [24]. Many of these studies have also established known group validity to self-reported health or chronic disease, but emphasised the need to establish the psychometric properties of these measures in different clinical populations. Both PedsQl and CHU9D have been used as PROMs in clinical trials of obesity prevention and treatment, yet only two studies [15, 25] have investigated the psychometric properties of these measures in the context of overweight and obesity. These studies in the UK and China found that among children aged 5–6 years, neither PedsQL nor CHU9D discriminated between children with healthy weight and those with overweight or obesity. The importance of re-evaluating the psychometric properties of HRQoL measures in different clinical populations has been noted [12, 21] as has the importance of evaluating responsiveness to change in health over time [26, 27]. A recent review of the psychometric performance of utility-based HRQoL instruments [28] highlighted that good psychometric performance in a general population would not necessarily indicate good performance in specific clinical conditions. Thus, it is important that psychometric assessment is conducted in the population of interest, with age, health condition and context likely to influence the performance of an instrument. Given the gap in our current knowledge, the aim of the present study was to assess the psychometric properties, including reliability, acceptability, validity and responsiveness of the PedsQL and the CHU9D in the measurement of HRQoL among children and adolescents in different weight status groups. We hypothesised that good psychometric performance would be shown across children in different weight status groups.

## Methods

### Participants

Data used in this study were from 6544 child participants of the Longitudinal Study of Australian Children (LSAC) [29]. The LSAC is an ongoing, large population survey of children and their families, that collects data on child development and wellbeing. It uses a stratified sampling design and is designed to be representative of the Australian child population. The LSAC recruited two cohorts of children in 2004 using clustered sampling methods: 5107 children in the Birth (B) cohort and 4983 children in the Kindergarten (K) cohort [30]. Children and their caregivers were interviewed every 2 years, with the most recent wave of data collection in 2020. In the present study, we used all longitudinal data from both the B and K cohorts in which both PedsQL and CHU9D were included. This covered the ages of 10–17 years and encompassed waves 6, 7 and 8 of the B cohort, in which children were aged 10/11, 12/13 and 14/15 years, and waves 6 and 7 of the K cohort in which children were aged 14/15 and 16/17 years.

### HRQoL measures

#### PedsQL

Generic health-related quality of life was measured using age-appropriate versions of the PedsQL v4.0 Generic Core Scales [31], with parent proxy report, hereafter referred to as PedsQL. From age 10 to 12 years, the ‘Parent report for Children’ was used and from 13 to 17 years the ‘Parent report for Teens’ was used. The PedsQL consists of 23 questions covering domains of physical, emotional, social and school functioning. Each item is scored on a 5-point scale (0 = never a problem; 1 = almost never a problem; 2 = sometimes a problem; 3 = often a problem; 4 = almost always a problem) and then reverse transformed, such that the Total Scale Score represents the sum of scores across all 23 items and ranges from 0 to 100, with higher scores indicating better HRQoL. Summary scores for Physical functioning, Emotional functioning, Social functioning and School functioning can also be calculated from subsets of the 23 items.

#### CHU9D

The CHU9D is a preference-based health-related quality of life measure, developed with children and validated for a target age of 7–11 years. It has also been validated for use amongst a general population of adolescents, aged 11–17 years [23, 32, 33]. It comprises 9 dimensions: worry, sadness, pain, tiredness, annoyance, school, sleep, daily routine and activities; each of which are scored at 5 levels of difficulty, self-reported by the child. The CHU9D has been valued in several different country contexts, which enables the calculation of utility scores used for estimating QALYs in economic evaluations. In the LSAC data, utilities from the CHU9D were determined using the Australian valuation algorithm developed in adolescents [32] and take the possible range of values from −0.1059 (poorest health) to 1 (perfect health).

### Weight status

At each wave of data collection in the LSAC, consenting children had their height and weight measured by trained research assistants. Height was measured with a laser stadiometer and weight was measured with Tanita body fat scales [30]. From height and weight, we calculated BMI-z scores (BMI-z) according to WHO standards [34]. Weight status was determined from BMI-z using the following definitions: healthy and underweight: BMI-z < 1; overweight: BMI-z ≥ 1 and <2; obesity: BMI-z ≥ 2. The proportion of child records in the underweight category (BMI-z < −1) was extremely low (<1%) so they were included with healthy weight. BMI-z values >5 and <−5 (*n* = 15) were dropped from the analyses as these are considered biologically implausible [35].

### Demographic characteristics

Age (in years), sex (male or female), socioeconomic position (SEP) (High or Low), culturally and linguistically diverse (CALD) status (CALD/not CALD) and Indigenous status (Aboriginal or Torres Strait Islander) were included as controls in the analyses. Individual-level socioeconomic position was measured at each wave using a variable developed by the LSAC study investigators which combined the education level, occupation type and income of the child’s caregivers into a *z*-score [36]. For simplicity, we categorised this variable into high (SEP *z*-score ≥ 0) and low SEP (SEP *z*-score < 0). A language other than English regularly spoken to the child, collected at age 2–3 years for the B cohort and age 4–5 years for the K cohort, was used as a proxy for CALD status.

### Psychometric properties/statistical analyses

The analyses of psychometric properties were conducted in accordance with practice guidelines and criteria for psychometric assessment [27, 37, 38]. For all analyses, except those assessing acceptability through missing data, we used observations that were complete for BMI, PedsQL, and CHU9D.

*Reliability* The only aspect of reliability we were able to assess with our existing dataset was internal consistency reliability, which is the degree of interrelatedness among items from the same scale [37]. Cronbach’s alpha and item-total correlations were used to assess the interrelation of the relevant individual items of PedsQL with the four summary scores and with the total score, and for the individual items of CHU9D with the total utility score scale, among children with overweight and obesity. A Cronbach’s alpha value ≥0.7 and item-total correlations ≥0.2 are considered acceptable thresholds for internal reliability consistency [38, 39].

*Acceptability* measures the quality of the data and is assessed by the completeness of the data and score distributions, including floor and ceiling effects. Acceptability may also include the practicality and feasibility of using a particular instrument among children with overweight and obesity, and may include measures of comprehension or burden of completion. Without access to respondents, we investigated acceptability through the assessment of missing data and the proportion of ceiling and floor values for the PedsQL total scores and CHU9D utility scores [40] across age and weight status. A low and acceptable level of missing data <5% was used as a benchmark [41], and the threshold for the acceptable floor and ceiling values was <10% [40].

*Validity* was addressed through known groups validity and convergent validity. *Known groups validity* is the extent to which a HRQoL measure can distinguish groups of children with and without a health condition, or between children with different severity of a condition. We hypothesised that children with higher weight status would have lower HRQoL and investigated known groups validity using general estimating equations (GEE) to account for the repeated measures of weight status and HRQoL among the same children, with adjustment for socio-demographic characteristics known to impact on HRQoL [39]. The GEE models included binomial family, log-link function and robust variance estimation. PedsQL Total Scale Scores (transformed to 0–1 scale) and CHU9D utility scores were the response variables; explanatory variables were weight status (healthy, overweight, obesity) and demographic variables, as described above. Interaction terms of weight status and significant demographic variables were included to identify whether these parameters modified the association of HRQoL and weight status. Models were fitted separately for girls and boys and significance levels were set at *p* < 0.05 for main effects and *p* < 0.01 for interaction terms (Wald tests). The *margins* command in STATA was used to predict marginal effects of weight status on reduced HRQoL, and to predict HRQoL by age and weight status, using final models including interaction terms, where significant.

C*onvergent validity* measures the level of agreement between instruments that purport to measure the same construct, and usually uses an existing health measure as a comparator. As PedsQL and CHU9D are well established and accepted measures of the same general construct i.e. HRQoL, we assessed convergent validity by calculating Spearman’s correlations between the CHU9D utility scores and the PedsQL Total Scale Score among children in each weight status group. Correlation coefficients >0.8 are regarded as strong, between 0.61 and 0.8 as good, between 0.41 and 0.6 as moderate and <0.4 as weak convergent validity [28]. We hypothesised that there would be moderate correlation between the two instruments, as they are both measures of HRQoL, but one is child report and the other is parent proxy.

*Responsiveness* is the ability of a measure to detect change over time when there are known changes in health status. [42]. This was examined by whether changes in the PedsQL total score and CHU9D utility score were responsive to changes in weight status between subsequent waves in the LSAC. Children were classified as to whether their weight status stayed the same, improved or deteriorated between consecutive waves of LSAC, according to the three weight status groups: healthy, overweight or obese. Both the B and K cohorts were used in the analysis, providing data on the change in HRQoL scores for individual children over 2-year intervals from mean ages 11–13, 13–15 and 15–17 years. We hypothesised that deterioration in weight status (healthy to overweight; healthy to obese; overweight to obese) would result in a negative HRQoL score change, whilst improvement in weight status (overweight to healthy; obese to overweight; obese to healthy) would result in a positive change in HRQoL scores, and no change in weight status would result in a HRQoL score change close to zero. Standardised response means (SRM) and effect sizes (ES), which take into account the change in HRQoL score in relation to the SD of baseline score, were calculated according to the method outlined in [43].

## Results

### Participants

Descriptive statistics of the analysis population are shown in Table 1. Across all ages and cohort groups, a total of 15,166 records from 6544 children were available for analysis. The distribution of demographic characteristics varied across weight groups, with a higher proportion of boys, children at low SEP, and children from linguistically diverse or Indigenous families having obesity compared with the healthy weight and overweight categories. At all ages and cohorts, mean PedsQL scores decreased with higher weight status. For the CHU9D, mean scores decreased with higher weight status at age 14–15 years in the B and K cohorts and 16–17 years in the K cohort, but not among children 10/11 and 12/13 years.

**Table 1:** Descriptive characteristics of analysis population by weight status and age.

B Cohort		Healthy weight	Overweight	Obesity
10–11 years (*N* = 3408)	*N* (%)	2266 (67)	752 (22)	390 (11)
	Female *n* (%)	1147 (51)	385 (51)	137 (35)
	Low socioeconomic position *n* (%)	1068 (47)	409 (54)	262 (67)
	Indigenous status *n* (%)	54 (2.3)	17 (2.3)	14 (3.6)
	Culturally diverse *n* (%)	368 (16)	143 (19)	74 (19)
	PedsQL mean (SD)	81.4 (12.7)	80.2 (13.3)	75.8 (15.0)
	CHU9D mean (SD)	0.80 (0.17)	0.79 (0.18)	0.80 (0.18)
12–13 years (*N* = 3071)	*N* (%)	2081 (68)	690 (22)	300 (10)
	Female *n* (%)	1008 (48)	360 (52)	118 (39)
	Low socioeconomic position *n* (%)	974 (47)	368 (53)	199 (66)
	Indigenous status *n* (%)	45 (2.2)	17 (2.5)	13 (4.3)
	Culturally diverse *n* (%)	346 (17)	126 (18)	58 (19)
	PedsQL mean (SD)	82.1 (13.0)	79.7 (13.8)	75.6 (15.3)
	CHU9D mean (SD)	0.82 (0.18)	0.80 (0.19)	0.81 (0.18)
14–15 years (*N* = 2831)	*N* (%)	1968 (70)	597 (21)	266 (9)
	Female *n* (%)	930 (47)	320 (54)	113 (42)
	Low socioeconomic position *n* (%)	910 (46)	314 (53)	180 (68)
	Indigenous status *n* (%)	39 (2.0)	11 (1.8)	11 (4.1)
	Culturally diverse *n* (%)	339 (17)	114 (19)	50 (19)
	PedsQL mean (SD)	80.3 (15.3)	78.2 (15.5)	72.8 (17.9)
	CHU9D mean (SD)	0.81 (0.19)	0.79 (0.21)	0.77 (0.21)
*K cohort*				
14–15 years (*N* = 3136)	*N* (%)	2192 (70)	632 (20)	312 (10)
	Female *n* (%)	1060 (48)	331 (52)	127 (41)
	Low socioeconomic position *n* (%)	1035 (47)	333 (53)	209 (67)
	Indigenous status *n* (%)	33 (1.5)	21 (3.3)	13 (4.2)
	Culturally diverse *n* (%)	363 (17)	109 (17)	62 (20)
	PedsQL mean (SD)	81.0 (14.7)	78.2 (15.8)	72.6 (18.3)
	CHU9D mean (SD)	0.81 (19.3)	0.79 (0.22)	0.76 (0.23)
16–17 years (*N* = 2720)	*N* (%)	1902 (70)	525 (19)	293 (11)
	Female *n* (%)	909 (48)	264 (50)	141 (48)
	Low socioeconomic position *n* (%)	880 (46)	296 (56)	184 (63)
	Indigenous status *n* (%)	24 (1.3)	14 (2.7)	14 (4.8)
	Culturally diverse *n* (%)	323 (17)	90 (17)	60 (20)
	PedsQL mean (SD)	82.1 (13.3)	79.9 (14.4)	74.8 (16.8)
	CHU9D mean (SD)	0.79 (0.21)	0.77 (0.22)	0.77 (0.24)

### Psychometric properties

#### Internal consistency

Among children and adolescents with overweight and obesity, internal consistency was strong for the PedsQL total score scale and the individual summary score subscales for physical health and emotional, social and school functioning (Cronbach’s alpha ranged from 0.77 to 0.92 and item-total correlations ranged from 0.40 to 0.77). CHU9D utility scores also showed strong internal consistency (Cronbach’s alpha 0.82 and item-total correlations ranged from 0.40 to 0.62) (supplementary Table 1).

*Acceptability* of the two measures was high, based on the overall low level of missing PedsQL scores of 1.6–2.0% and missing CHU9D utility of 1.3–1.6% (supplementary Table 2). Examination of missing data across age and weight status groups, also indicated an acceptable level of missing values <5% for both PedsQL and CHU9D. No floor effect was observed for the PedsQL and floor effects for the CHU9D were <0.1%. There were no ceiling effects for PedsQL, but >10% of children scored at full health (=1) on the CHU9D, which is normal and acceptable for a preference-based measure (i.e. one providing utilities) [44].

#### Known groups

The PedsQL was able to discriminate between children with overweight and obesity compared to those in healthy weight (Table 2). After adjustment for demographic factors and compared with healthy weight, the differences in marginal predictions of PedsQL score for boys and girls with obesity were: boys −5.6 (95%CI −6.7, −4.4), *p* < 0.001; girls −6.7 (95%CI −8.1, −5.4), *p* < 0.001, and for those with overweight: boys −2.2 (95%CI −3.0, −1.4), *p* < 0.001; girls −1.3 (95%CI −2.0, −0.5), *p* = 0.002. The PedsQL also indicated known groups validity for boys and girls from low compared to high SEP (*p* < 0.001) and from CALD compared to non-CALD households (*p* < 0.001). All interaction terms investigated were non-significant (*p* > 0.01), indicating no evidence that age or demographic characteristics modifies the relationship between PedsQL score and weight status (see supplementary Table 3).

**Table 2:** Association of CHU9D utility and PedsQL total score with weight status, using general estimating equations with binomial log link, and adjustment for age, Indigenous status, cultural diversity and socio-economic position.

	Characteristic	PedsQL		CHU9D	
		Mean total score coefficient (95% CI)	*p*	Mean utility score coefficient (95% CI)	*p*
*Boys*	Weight status				
	Healthy weight	Referent		Referent	
	Overweight	−0.027 (−0.037, −0.017)	<0.001	−0.010 (−0.023, 0.003)	0.14
	Obesity	−0.070 (−0.086, −0.054)	<0.001	−0.025 (−0.043, −0.006)	0.008
	Age	0.001 (0.000, 0.003)	0.16	0.008 (0.006, 0.011)	<0.001
	Indigenous status	−0.012 (−0.051, −0.027)	0.531	0.045 (0.014, 0.076)	0.005
	Culturally diverse	−0.043 (−0.058, −0.028)	<0.001	0.0055 (−0.010, 0.021)	0.479
	Low socioeconomic position	−0.031 (−0.040, −0.022)	<0.001	0.006 (−0.005, 0.017)	0.313
	Constant	−0.188 (−0.211, −0.165)	<0.001	0.301 (0.336, 0.266)	<0.001
Girls	Weight status				
	Healthy weight	Referent		Referent	
	Overweight	−0.016 (−0.026, −0.006)	0.002	−0.019 (−0.035, −0.002)	0.027
	Obesity	−0.088 (−0.108, −0.069)	<0.001	−0.046 (−0.073, −0.019)	0.001
	Age	−0.005 (−0.006, −0.003)	<0.001	−0.016 (−0.018, −0.013)	<0.001
	Indigenous status	−0.096 (−0.049, 0.030)	0.63	0.005 (−0.041, 0.051)	0.82
	Culturally diverse	−0.044 (−0.059, −0.028)	<0.001	−0.008 (−0.027, 0.010	0.374
	Low socioeconomic position	−0.021 (−0.030, −0.012)	<0.001	−0.001 (−0.018, 0.009)	0.554
	Constant	−0.143 (−0.167, 0.120)	<0.001	−0.036 (−0.074, 0.001)	0.059

Similarly, CHU9D utility scores were lower for boys and girls with obesity compared to those with healthy weight (Table 2). Differences in marginal predictions of CHU9D for obesity compared with healthy weight were: boys −0.02 (95%CI −0.034, −0.006), *p* < 0.002; girls −0.035 (95%CI −0.054, −0.015), *p* = 0.001. However, for those with overweight, only among girls was there a statistically significant difference in CHU9D utility score compared to healthy weight: girls −0.014 (95%CI −0.026, −0.003), *p* = 0.02); boys −0.008 (95%CI −0.018, 0.002), *p* = 0.146. CHU9D utility scores declined with increasing age for girls (*p* < 0.001), but did not discriminate between groups of culturally diverse, indigenous or socioeconomically disadvantaged children. Interaction terms between family demographic factors and weight status were not significant (*p* > 0.01), indicating similar utility score differences by weight status, for indigenous children and those from low SEP and CALD groups. However, a significant interaction between age and obesity for girls (*p* = 0.004) indicated that utilities decline with age, but they decline faster for girls with obesity than for those in healthy weight (supplementary Table 4).

**Fig. 1:**
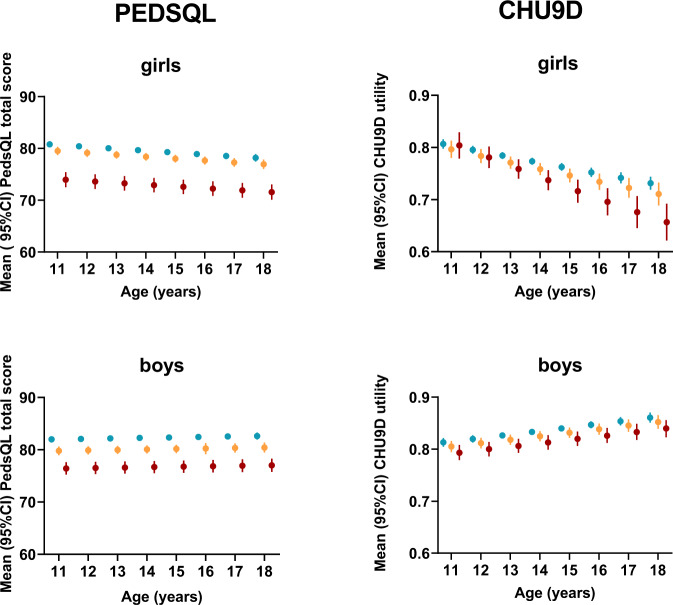
Margins predictions of PedsQL total score and CHU9D utility score by age and weight status from adjusted models. Red = obesity; orange = overweight; blue = healthy weight.

Marginal predictions for final models (including interaction terms where significant) for CHU9D utility and PedsQL total score by age and weight status, depict the *age-independent* association of weight status and PedsQL score, and the *age-dependent* association of CHU9D utility for girls and the stronger decline in HRQoL for those affected by obesity (Fig. 1). For example, for girls aged 12 years, the predicted CHU9D utility decrement for obesity was 0.015, but at age 17 was 0.065. A rule of thumb for the minimal clinically meaningful difference of utility scores of 0.03 [45] was exceeded with CHU9D for girls with obesity aged 14 and above, but not for boys or for overweight. For PedsQL, the clinically meaningful difference of 4.5 points [18] was exceeded for obesity compared to healthy weight for girls and boys across all ages, but not for overweight.

#### Convergent validity

In the tests of convergent validity between PedsQL and CHU9D by weight status and age, the Spearman correlation coefficients ranged from 0.16 to 0.29, which is considered low evidence of convergence between the two instruments (supplementary Table 5).

#### Responsiveness

**Fig. 2:**
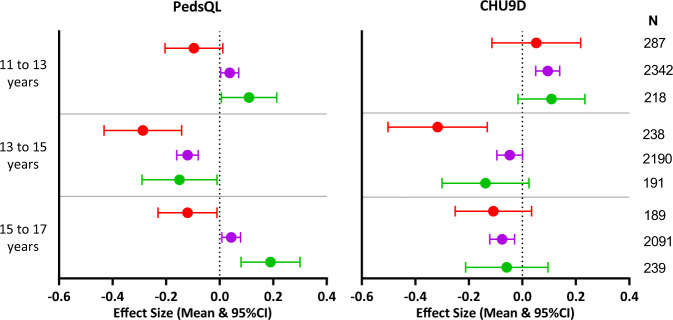
Responsiveness, measured by effect size of changes in PedsQL total score and CHU9D utility score in response to changes in weight status over childhood and adolescence. Green = improvement in weight status; purple = no change in weight status; red = worsening weight status.

The effect sizes of PedsQL were mostly consistent with the hypothesised direction according to change in weight status (Fig. 2), producing a positive ES for ‘better’, close to 0 for ‘same’ and negative ES for ‘worse’ weight status change. CHU9D ES were less consistent with the hypothesis, for example between 11 and 13 years, all CHU9D ES were positive regardless of the direction of change in weight status, and among 15–17 years all ES were negative, regardless of actual weight change. Between 13 and 15 years, the pattern was less consistent for both measures, with negative ES for all changes in weight status, including the ‘same’ category and the ‘better’ category. In addition to greater consistency in the hypothesised direction of change, PedsQL ES were larger than for the CHU9D, although all ES were relatively small.

## Discussion

Two paediatric HRQoL instruments have been investigated in the context of their application to child and adolescent weight status, focusing on reliability, acceptability, validity and responsiveness. This is the first time that rigorous psychometric assessment of PedsQl and CHU9D has been carried out for children with overweight and obesity in this age range. Internal consistency reliability was demonstrated for the PedsQL and the CHU9D, and both measures had high acceptability based on the low proportion of floor values. There were no ceiling effects for the PedsQL, but >10% of children scored at full health (=1) on the CHU9D, which is normal and acceptable for a preference-based measure. Based on the low number of missing responses, both PROMs were acceptable for the measurement of HRQoL in children and adolescents with overweight and obesity. We were unable to investigate patient burden with our dataset, but this aspect of acceptability and patient comprehension remain important features to assess in future studies.

The PedsQL demonstrated very good known group validity, with the ability to discriminate between groups of children based on weight status, and between those from lower sociodemographic and culturally diverse households. The CHU9D showed good discrimination between obesity and healthy weight, but was unable to discriminate between weight categories in younger girls and between overweight and healthy weight in boys. This may be because the CHU9D questions are not sensitive enough to pick up on the changes in HRQoL associated with weight-related co-morbidities in younger children and boys.

Known group validity is an important property for the measurement of patient HRQoL in trials of obesity prevention, with intervention effectiveness contingent on the instrument having the ability to detect differences between weight status groups. There is no existing literature on children and adolescents aged 11–17 years for direct comparison with our results, but two previous studies with younger children aged 5–6 years found that PedsQL and CHU9D had poor known group validity with respect to weight status. [15, 25]. The differences between these studies and our study are most likely a result of the different age groups studied.

PedsQL had quite consistent quality of life scores associated with overweight and obesity in girls and boys and across all ages studied. The age-related decline of CHU9D utility, particularly among girls with obesity has been noted before [14] and resulted in quite different CHU9D utility scores among boys and girls living with obesity, particularly in late adolescence. The implications for cost-utility analyses using this measure, are that similar weight status among girls and boys would lead to different utilities and different QALYs, and thus poorer cost-effectiveness in boys compared with girls. In addition, the CHU9D did not discriminate between boys with overweight and those with healthy weight, which may limit its application among boys, when using the CHU9D in economic evaluations of prevention and treatment of overweight. These limitations may partly explain the paucity of economic evaluations of childhood obesity prevention, which remains a major challenge [46].

Our investigation of convergent validity found low correlation between the PedsQL total scores and the CHU9D utility score, despite the fact that they are measuring the same construct. The low correlation may be explained by a number of points of difference in the LSAC data: PedsQL was proxy report and the CHU9D was self-report, they have different numbers of items: 23 and 9, respectively, and different scoring systems. The PedsQL total score is a summative average of the reverse scored items of the respondents, whilst for the CHU9D a preference-based value set/algorithm is applied to the respondents’ item scores to calculate the CHU9D utility score.

Responsiveness is an important quality of HRQoL measures in health [42] that has rarely been assessed among children and adolescents [28] and never before in the context of overweight and obesity. We found the PedsQL to be responsive to changes in weight status in that the effect size and standardised response means were consistent with the hypothesised direction, while the CHU9D was less responsive. This suggests that the PedsQL would be an appropriate instrument to use in obesity management and prevention, but there may be some limitations in using the CHU9D due to lower responsiveness. The better validity and responsiveness of the PedsQL over the CHU9D may be due to the larger number of items, or because the questions themselves are more relevant to the impacts of obesity.

### Strengths and weaknesses

Strengths of this study include the size and richness of the dataset (*n* = 15,166 data points), and its longitudinal nature which allowed us to investigate responsiveness to weight change which has not previously been evaluated. Overweight and obesity were based on objectively measured height and weight and thus not subject to reporting bias. The psychometric methods used were rigorous and based on established gold standard criteria. There are some weaknesses: as mentioned, we were unable to investigate patient burden and comprehension from our established dataset. In addition, the 2-year interval of data collection could not assess test-retest reliability. Another potential limitation is that we were only able to evaluate the parent proxy report of PedsQL, as the child self-report version was not used in the LSAC. Previous studies [47] however, have found very similar HRQoL scores for proxy and child report, but nevertheless it may impact comparison with the CHU9D which is child/adolescent self-reported measure.

While this study explores the psychometric properties of PedsQL and CHU9D across children in different weight status groups, defined by BMI-z, future research may consider repeating these analyses using other measures of child growth, such as waist circumference, or using co-morbidities of overweight and obesity such as asthma and depression. Another important area of future research would be to assess psychometric performance of the two measures in further paediatric clinical conditions to ascertain whether the poorer responsiveness and validity of the CHU9D is specific to obesity or a more general feature of this measure.

## Conclusion

Overall, both PROMs demonstrated adequate reliability and acceptability for the measurement of HRQoL in children and adolescents with overweight or obesity. However, PedsQL appears to be superior to the CHU9D in terms of its ability to discriminate between children of different weight status and to respond to changes in weight status over time. This represents a dilemma in cost-utility analysis of overweight and obesity prevention and treatment, as the CHU9D is unlikely to be sensitive enough to detect improvements in weight status. Evidence of value for money based on QALYs underpins decision making by health technology assessment agencies in many jurisdictions around the world, thus the psychometric properties of preference based HRQoL measures are vitally important.

## Supplementary information

Supplemental material

## Data Availability

The data from LSAC used in this study are available by application to the data custodians: Longitudinal Studies, Data Strategy Branch, Australian Government Department of Social Services.

## Code availability

STATA code for the analysis is available from the authors on request.

## References

[1] Ng M, Fleming T, Robinson M, Thomson B, Graetz N, Margono C (2014). Global, regional, and national prevalence of overweight and obesity in children and adults during 1980-2013: A systematic analysis for the Global Burden of Disease Study 2013. Lancet..

[2] Skinner AC, Perrin EM, Moss LA, Skelton JA (2015). Cardiometabolic risks and severity of obesity in children and young adults. N Engl J Med.

[3] Hannon TS, Rao G, Arslanian SA (2005). Childhood obesity and type 2 diabetes mellitus. Pediatrics..

[4] Deng X, Ma J, Yuan Y, Zhang Z, Niu W (2019). Association between overweight or obesity and the risk for childhood asthma and wheeze: An updated meta-analysis on 18 articles and 73,252 children. Pediatr Obes.

[5] Smith SM, Sumar B, Dixon KA (2014). Musculoskeletal pain in overweight and obese children. Int J Obes.

[6] Rao WW, Zong QQ, Zhang JW, An FR, Jackson T, Ungvari GS (2020). Obesity increases the risk of depression in children and adolescents: Results from a systematic review and meta-analysis. J Affective Disord.

[7] Tsiros MD, Olds T, Buckley JD, Grimshaw P, Brennan L, Walkley J (2009). Health-related quality of life in obese children and adolescents. Int J Obes.

[8] Killedar A, Lung T, Petrou S, Teixeira-Pinto A, Tan EJ, Hayes A (2020). Weight status and health-related quality of life during childhood and adolescence: effects of age and socioeconomic position. Int J Obes.

[9] Ul-Haq Z, MacKay DF, Fenwick E, Pell JP (2013). Meta-analysis of the association between body mass index and health-related quality of life among children and adolescents, assessed using the pediatric quality of life inventory index. J Pediatr.

[10] Kwon J, Freijser L, Huynh E, Howell M, Chen G, Khan K (2022). Systematic Review of Conceptual, Age, Measurement and Valuation Considerations for Generic Multidimensional Childhood Patient-Reported Outcome Measures. Pharmacoeconomics.

[11] Arsiwala T, Afroz N, Kordy K, Naujoks C, Patalano F (2021). Measuring What Matters for Children: A Systematic Review of Frequently Used Pediatric Generic PRO Instruments. Ther Innov Regul Sci.

[12] Stevens K (2011). Assessing the performance of a new generic measure of health-related quality of life for children and refining it for use in health state valuation. Appl Health Econ Health Policy.

[13] Ahuja B, Klassen AF, Satz R, Malhotra N, Tsangaris E, Ventresca M (2014). A review of patient-reported outcomes for children and adolescents with obesity. Qual Life Res.

[14] Killedar A, Lung T, Petrou S, Teixeira-Pinto A, Hayes A (2020). Estimating Age- and Sex-Specific Utility Values from the CHU9D Associated with Child and Adolescent BMI z-Score. Pharmacoeconomics..

[15] Frew EJ, Pallan M, Lancashire E, Hemming K, Adab P (2015). Is utility-based quality of life associated with overweight in children? Evidence from the UK WAVES randomised controlled study. BMC Pediatr.

[16] Eminson K, Canaway A, Adab P, Lancashire E, Pallan M, Frew E (2018). How does age affect the relationship between weight and health utility during the middle years of childhood?. Qual Life Res.

[17] Baile JI, Guevara RM, Gonzalez-Calderon MJ, Urchaga JD (2020). The Relationship between Weight Status, Health-Related Quality of Life, and Life Satisfaction in a Sample of Spanish Adolescents. Int J Environ Res Public Health.

[18] Varni JW, Burwinkle TM, Seid M, Skarr D (2003). The PedsQL™* 4.0 as a pediatric population health measure: Feasibility, reliability, and validity. Ambul Pediatr.

[19] Engelen V, Haentjens MM, Detmar SB, Koopman HM, Grootenhuis MA (2009). Health related quality of life of Dutch children: psychometric properties of the PedsQL in the Netherlands. BMC Pediatr.

[20] Gkoltsiou K, Papaevangelou V, Tountas Y, Constantopoulos A (2008). Measuring quality of life in Greek children: First psychometric results of the Greek version of the pediatric quality of life inventory (PEDSQL) 4.0 generic core scales. Pediatrics..

[21] Petersen KD, Ratcliffe J, Chen G, Serles D, Frosig CS, Olesen AV (2019). The construct validity of the Child Health Utility 9D-DK instrument. Health Qual Life Outcomes.

[22] Lindvall K, Vaezghasemi M, Feldman I, Ivarsson A, Stevens KJ (2021). Petersen S. Feasibility, reliability and validity of the health-related quality of life instrument Child Health Utility 9D (CHU9D) among school-aged children and adolescents in Sweden. Health Qual Life Outcomes.

[23] Stevens K, Ratcliffe J (2012). Measuring and valuing health benefits for economic evaluation in adolescence: An assessment of the practicality and validity of the child health utility 9d in the australian adolescent population. Value Health.

[24] Yang P, Chen G, Wang P, Zhang K, Deng F, Yang H (2018). Psychometric evaluation of the Chinese version of the Child Health Utility 9D (CHU9D-CHN): a school-based study in China. Qual Life Res.

[25] Zanganeh M, Adab P, Li B, Frew E (2021). An assessment of the construct validity of the Child Health Utility 9D-CHN instrument in school-aged children: evidence from a Chinese trial. Health Qual Life Outcomes.

[26] Furber G, Segal L (2015). The validity of the Child Health Utility instrument (CHU9D) as a routine outcome measure for use in child and adolescent mental health services. Health Qual Life Outcomes.

[27] Mokkink LB, Terwee CB, Patrick DL, Alonso J, Stratford PW, Knol DL (2010). The COSMIN checklist for assessing the methodological quality of studies on measurement properties of health status measurement instruments: An international Delphi study. Qual Life Res.

[28] Rowen D, Keetharuth AD, Poku E, Wong R, Pennington B, Wailoo A (2021). A Review of the Psychometric Performance of Selected Child and Adolescent Preference-Based Measures Used to Produce Utilities for Child and Adolescent Health. Value Health.

[29] Sanson A, Australian Institute of Family Studies. Introducing the Longitudinal Study of Australian Children. Melbourne. 2022. http://www.aifs.gov.au/lsac/pubs/discussionpaper1.pdf.

[30] Mohal JLC, Howell L, Renda J, Jessup K, Daraganova G. Growing Up in Australia: The Longitudinal Study of Australian Children – Data User Guide, Release 8.0, October 2020. Melbourne: Australian Institute of Family Studies; 2020.

[31] Varni JW, Seid M, Kurtin PS (2001). PedsQL™ 4.0: Reliability and Validity of the Pediatric Quality of Life Inventory™ Version 4.0 Generic Core Scales in Healthy and Patient Populations. Med Care.

[32] Ratcliffe J, Huynh E, Chen G, Stevens K, Swait J, Brazier J (2016). Valuing the Child Health Utility 9D: Using profile case best worst scaling methods to develop a new adolescent specific scoring algorithm. Soc Sci Med.

[33] Ratcliffe J, Stevens K, Flynn T, Brazier J, Sawyer M (2012). An assessment of the construct validity of the CHU9D in the Australian adolescent general population. Qual Life Res.

[34] WHO. Growth reference data for 5-19 years. 2021. https://www.who.int/growthref/en/.

[35] WHO. Application tools. 2021. http://www.who.int/growthref/tools/en/.

[36] Baker K, Sipthorp M, Edwards B, Australian Institute of Family Studies, issuing body & Growing Up in Australia, the Longitudinal Study of Australian Children. A Longitudinal Measure of Socioeconomic Position in LSAC. Australian Institute of Family Studies; Melbourne, VIC, 2017. http://nla.gov.au/nla.obj-571991763.

[37] Reeve BB, Wyrwich KW, Wu AW, Velikova G, Terwee CB, Snyder CF (2013). ISOQOL recommends minimum standards for patient-reported outcome measures used in patient-centered outcomes and comparative effectiveness research. Qual Life Res.

[38] Brazier J, Deverill M (1999). A checklist for judging preference-based measures of health related quality of life: Learning from psychometrics. Health Econ.

[39] Von Rueden U, Gosch A, Rajmil L, Bisegger C, Ravens-Sieberer U (2006). Socioeconomic determinants of health related quality of life in childhood and adolescence: Results from a European study. J Epidemiol Community Health.

[40] Smith SC, Lamping DL, Banerjee S, Harwood R, Foley B, Smith P (2005). Measurement of health-related quality of life for people with dementia: Development of a new instrument (DEMQOL) and an evaluation of current methodology. Health Technol Assess.

[41] Little TD, Jorgensen TD, Lang KM, Moore EWG (2014). On the joys of missing data. J Pediatr Psychol.

[42] Mokkink LB, Terwee CB, Patrick DL, Alonso J, Stratford PW, Knol DL (2010). The COSMIN study reached international consensus on taxonomy, terminology, and definitions of measurement properties for health-related patient-reported outcomes. J Clin Epidemiol.

[43] Pink J, Petrou S, Williamson E, Williams M, Lamb SE (2014). Properties of patient-reported outcome measures in individuals following acute whiplash injury. Health Qual Life Outcomes.

[44] Richardson J, McKie J, Bariola, E. Multiattribute utility instruments and their use. In: Culyer AJ (Ed) Encyclopedia of Health Economics. Elsevier; 2014, pp 341–57.

[45] Michael F, Drummond MJS, Claxton K, Stoddart GL, Torrance GW. Methods for the Economic Evaluation of Health Care Programmes. 4th ed. Oxford: Oxford University Press; 2015.

[46] Frew E (2016). Economic Evaluation of Childhood Obesity Interventions: Reflections and Suggestions. Pharmacoeconomics..

[47] Williams JWCL, Hesketh KD, Hardy P, Waters EB, Patton GC, Wake M. Changes in body mass index and health related quality of life from childhood to adolescence. Int J Pediatr Obes. 2011;6:e442–8. 10.3109/17477166.2010.526226 21198354

