# Supplementary material for: Reliability, acceptability, validity and responsiveness of the CHU9D and PedsQL in the measurement of quality of life in children and adolescents with overweight and obesity
Source: Int J Obes (Lond). 2023 Apr 18;47(7):622–9. doi: 10.1038/s41366-023-01305-5 (PMC10299908; doi:10.1038/s41366-023-01305-5)
Supplement: Supplementary file 1 — Supplemental material [file 41366_2023_1305_MOESM1_ESM.pdf]

Supplementary appendix This supplementary file contains supplementary tables referred to in *Hayes et al., Reliability, acceptability, validity and responsiveness of the CHU9D and PedsQL in the measurement of quality of life in children and adolescents with overweight and obesity.*

Supplementary Table 1 Internal consistency results for PedsQL and CHU9D among children and adolescents in different weight status groups

**Condition**	**Variable**	**Cronbach's alpha value [a]**	**Item-total correlations range [b]**
Healthy weight			
	PedsQL Physical health summary score subscale	0.8602	0.3778 - 0.7769
	PedsQL Emotional functioning summary score subscale	0.8260	0.5572 - 0.6922
	PedsQL Social functioning summary score subscale	0.8144	0.5530 - 0.6630
	PedsQL School functioning summary score subscale	0.7736	0.4346 - 0.6537
	PedsQL Total score	0.9132	0.4634 - 0.6170
	CHU9D utility score	0.7951	0.3736 - 0.6087
Overweight			
	PedsQL Physical health summary score subscale	0.8568	0.3999 - 0.7720
	PedsQL Emotional functioning summary score subscale	0.8397	0.5719 - 0.7212
	PedsQL Social functioning summary score subscale	0.8207	0.5677 - 0.6732
	PedsQL School functioning summary score subscale	0.7666	0.4309 - 0.6361
	PedsQL Total score	0.9139	0.4440 - 0.6081
	CHU9D utility score	0.8152	0.4005 - 0.6212
Obesity			
	PedsQL Physical health summary score subscale	0.8629	0.4321 - 0.7634
	PedsQL Emotional functioning summary score subscale	0.8449	0.5822 - 0.7285
	PedsQL Social functioning summary score subscale	0.8283	0.5660 - 0.6938
	PedsQL School functioning summary score subscale	0.7736	0.4451 - 0.6618
	PedsQL Total score	0.9228	0.4524 - 0.6682
	CHU9D utility score	0.8117	0.3976 - 0.5946

The sample to test internal consistency of PedsQL and CHU9D includes complete cases for weight status, PedsQL total score, CHU9D utility score (CHU9D), socioeconomic position, cultural diversity and Indigenous status, n=15,166 data points. [a] Acceptable Cronbach's alpha threshold ≥ 0.7; [b] Acceptable Item-total correlations threshold ≥ 0.2

Supplementary Table 2. Missing data and floor and ceiling effects for PedsQL and CHU9D in LSAC data set by weight status and age group

	B cohort 10-11 years old		B cohort 12-13 years old		B cohort 14-15 years old		K cohort 14-15 years old		B cohort 16-17 years old	
	N	Missing (%)	N	Missing (%)	N	Missing (%)	N	Missing (%)	N	Missing (%)
**1. PedsQL total score-missing data**										
Healthy weight	2 368	1.0	2 144	1.1	2 026	1.0	2 283	2.2	1 991	2.5
Overweight	782	1.3	713	0.7	618	1.5	663	2.4	552	2.9
Obese	412	1.0	312	1.3	282	1.4	324	3.1	317	3.5
**2. CHU9D utility score- missing data**										
Healthy weight	2 368	2.4	2 144	0.8	2 026	1.1	2 283	1.3	1 991	0.8
Overweight	782	1.2	713	1.5	618	1.3	663	1.7	552	0.9
Obese	412	1.7	312	1.3	282	3.2	324	0.6	317	1.6
**3. PedsQL total score-floor effects**										
Healthy weight	2 266	0	2 081	0	1 968	0	2 192	0	1 902	0
Overweight	752	0	690	0	597	0	632	0	525	0
Obese	390	0	300	0	266	0	312	0	293	0
**4. PedsQL total score-ceiling effects**										
Healthy weight	2 266	3.1	2 081	3.7	1 968	4.3	2 192	4.7	1 902	4.4
Overweight	752	3.3	690	3.8	597	4.5	632	3.8	525	2.9
Obese	390	1.5	300	3.0	266	1.9	312	4.2	293	2.4
**5. CHU9D utility score-floor effects**										
Healthy weight	2 266	<0.1	2 081	0	1 968	0	2 192	0	1 902	0
Overweight	752	0	690	0	597	0%	632	0.2	525	0
Obese	390	0	300	0	266	0%	312	0	293	0
**6. CHU9D utility score-ceiling effects**										
Healthy weight	2 266	10.9	2 081	15.6	1 968	20.2	2 192	17.4	1 902	22.0
Overweight	752	10.1	690	11.7	597	18.6	632	16.9	525	20.2
Obese	390	10.8	300	16.7	266	14.3	312	15.4	293	23.6

Supplementary Table 3 GEE binomial log (link) models for boys and girls PedsQL Total score including interaction terms between weight status and all other variables.

	Boys		Girls	
**Characteristic**	**PedsQL Total score**		**PedsQL Total score**	
	**coefficient (95% CI)**	**p**	**coefficient (95% CI)**	**p**
Overweight	0.007 (-0.048, 0.062)	0.802	0.030(-0.024, 0.084)	0.278
Obesity	-0.043 (-0.036, 0.123)	0.286	-0.035 (-0.134, 0.064)	0.134
Age (per year)	0.002 (-0.000, 0.004)	0.054	-0.004 (-0.006, -0.002)	**<0.001**
Indigenous status	-0.013 (-0.060, 0.035)	0.609	-0.017 (-0.064, 0.031)	0.490
Culturally diverse	-0.044 (-0.062, -0.027)	**<0.001**	-0.051 (-0.069, -0.033)	**<0.001**
Low SEP	-0.031 (-0.041, -0.021)	**<0.001**	-0.019 (-0.029, -0.008)	**<0.001**
Age # overweight	-0.003 (-0.007, 0.001)	0.186	-0.003 (-0.007, 0.000)	0.087
Age# obesity	-0.002 (-0.007, 0.004)	0.591	-0.003 (-0.010, 0.004)	0.395
Indigenous # overweight	0.029 (-0.053, 0.110)	0.487	-0.030 (-0.122, 0.062)	0.526
Indigenous # obesity	-0.065 (-0.165, 0.034)	0.198	0.094 (0.003, 0.184)	0.042
Culturally diverse # overweight	0.004 (-0.027, 0.035)	0.798	0.030 (0.001, 0.060)	0.046
Culturally diverse # obesity	0.010 (-0.033, 0.052)	0.659	0.008 (-0.044, 0.061)	0.754
Low SEP # overweight	0.004 (-0.016, 0.023)	0.688	-0.006 (-0.025, 0.012)	0.498
Low SEP # obesity	-0.010 (-0.040, 0.021)	0.536	-0.028(-0.063,0.007)	0.122
Constant	-0.197 (-0.223, -0.171)	**<0.001**	-0.156 (-0.183, -0.128)	**<0.001**

Bold =Significant variables at p<0.05 and significant interaction terms at p<0.01

Supplementary Table 4 GEE binomial log (link) models for boys and girls CHU9D utility score including interaction terms between weight status and all other variables.

Characteristic	Boys CHU9D utility score coefficient (95% CI)	p	Girls CHU9D utility score coefficient (95% CI)	p
Overweight	-0.016 (-0.102, 0.069)	0.713	0.008 (-0.086, 0.102)	0.871
Obesity	-0.038 (-0.147, 0.070)	0.489	0.143 (0.005, 0.282)	0.043
Age	0.008 (0.005, 0.0108)	**<0.001**	-0.014 (-0.017, -0.011)	**<0.001**
Indigenous status	0.062 (0.027, 0.097)	**<0.001**	-0.003 (-0.059, 0.053)	0.916
Culturally diverse	0.002 (- 0.169,0.021)	0.836	-0.009 (-0.030, 0.012)	0.399
Low SEP	0.005 (-0.008, 0.017)	0.485	-0.007 (-0.023, 0.009)	0.38
Age #overweight	0.000 (-0.006, 0.006)	0.994	-0.002 (-0.009, 0.005)	0.522
Age# obesity	0.001 (-0.0065, 0.009)	0.787	-0.015 (-0.025, -0.005)	**0.004**
Indigenous # overweight	-0.028 (-0.087, 0.030)	0.339	-0.007 (-0.138, 0.123)	0.911
Indigenous # obesity	-0.082 (-0.195, 0.031)	0.156	0.049 (-0.083, 0.180)	0.468
Culturally diverse # overweight	0.022 (-0.010, 0.021)	0.179	0.003 (-0.041, 0.046)	0.905
Culturally diverse # obesity	-0.005 (-0.031, 0.041)	0.839	-0.002 (-0.072, 0.069)	0.974
Low SEP # overweight	0.004 (-0.022, 0.031)	0.754	0.006 (-0.026, 0.038)	0.711
Low SEP # obesity	0.005 (-0.031, 0.041)	0.781	0.022 (-0.030, 0.074)	0.401
Constant	-0.299 (-0.340, -0.257)	**<0.001**	-0.056 (-0.010, -0.012)	**0.013**

Bold =Significant variables at p<0.05 and significant interaction terms at p<0.01

Supplementary Table 5. Convergent validity (Spearman correlation coefficients) between PedsQL (parent reported) and CHU9D (child reported) in LSAC data set by weight status and age group:

	PedsQL total score									
	**B cohort 10-11 years old**		**B cohort 12-13 years old**		**B cohort 14-15 years old**		**K cohort 14-15 years old**		**B cohort 16-17 years old**	
CHU9D utility score	N	Spearman correlation coefficient	N	Spearman correlation coefficient	N	Spearman correlation coefficient	N	Spearman correlation coefficient	N	Spearman correlation coefficient
**Healthy weight**										
	2 290	0.20	2 102	0.21	1 985	0.19	2 205	0.29	1 926	0.28
**Overweight**										
	763	0.16	698	0.22	602	0.24	638	0.23	531	0.29
**Obese**										
	402	0.21	304	0.19	270	0.26	312	0.25	302	0.28

All correlations significant ($p < 0.05$)
